# NSG2: a promising prognostic marker shaping the immune landscape of breast cancer

**DOI:** 10.3389/fimmu.2024.1487447

**Published:** 2024-10-18

**Authors:** Xuan Li, Qiming Gu, Pingping Sun, Lei Yang, Xiaojing Zhang, Bing Lu, Qichao Ni

**Affiliations:** ^1^ Department of General Surgery, Affiliated Hospital of Nantong University & Medical School of Nantong University, Nantong, China; ^2^ Clinical and Translational Research Center, Affiliated Hospital of Nantong University & Department of Oncology, Medical School of Nantong University, Nantong, Jiangsu, China

**Keywords:** NSG2, breast cancer, tumor microenvironment, prognosis, biomarker

## Abstract

**Background:**

Breast cancer (BC) remains a significant health issue globally and most common cause of mortality in women. Enhancing our understanding on biomarkers may greatly improve both diagnostic and therapeutic approaches to this disease.

**Methods:**

We retrospectively assessed tumor samples from 228 BC cases and 51 normal samples, alongside relevant clinical data. Neuronal vesicle trafficking associated 2(NSG2) expression was evaluated through bioinformatics and multiplex immunohistochemistry. Associations between NSG2 expression, tumor-infiltrating immune cells (TIICs), immune checkpoints, and clinical outcomes were investigated.

**Results:**

NSG2 was present in both breast cancer cells and adjacent stromal cells. Increased NSG2 expression in cancer cells correlated with greater tumor size, distant metastasis, and more advanced clinical stages. Kaplan-Meier survival and multivariate analyses identified NSG2 expression in both cancer and stromal cells as an independent prognostic factor for breast cancer survival. Elevated NSG2 levels both in cancer and stroma cells were linked to increased CD4+ T, CD8+ T, and Lamp3+ dendritic cells infiltration in stromal regions (*P* < 0.05). Conversely, the expression of NSG2 in the stroma was negatively correlated with CD20+ B cells (*P* < 0.05). Additionally, NSG2 expression was found to be associated with CTLA-4 levels (*P* < 0.05).

**Conclusion:**

NSG2 seems to be a significant component of the BC immune microenvironment and may serve as an important prognostic marker.

## 1 Introduction

Breast cancer (BC) is the most common cancer and a leading cause of cancer-related deaths in women worldwide (1, 2), with treatment decisions often based on molecular profiling of BC (3, 4). BC classification has traditionally been based on its receptors, and Her-2 expression to guide treatment choices (5). However, issues like drug resistance continue to pose significant challenges, underscoring the need for new strategies to enhance long-term patient outcomes (6).

Immune cells were traditionally considered inhibitors of cancer progression (7). However, emerging evidence reveals a more complex role for the immune microenvironment, which can both suppress tumor growth and facilitate tumor escape (8, 9). This evolving understanding has shifted focus towards immunotherapy as a promising strategy for enhancing patient outcomes (10). Tumor microenvironment (TME) is essential for cancer development and clinical prognosis (11). Analysis of tumor-infiltrating immune cells (TIICs) offers insights into mechanisms of immune evasion and opens avenues for novel therapeutic strategies (12). Therefore, integrating immune infiltration characteristics with molecular and histologic criteria is essential for advancing BC classification (13).

Immune checkpoint inhibitors can reverse the immune suppression induced by tumors, thereby restoring the immune system’s ability to target cancer cells (14), and have shown promise for patients who are resistant to conventional treatments or have poor prognoses.

Cancer neuroscience is an emerging field within cancer biology that seeks to elucidate the interactions between the nervous system, malignancy, and its microenvironment (15). Neural elements and BC exhibit a complex and interdependent relationship, with nerves significantly impacting patient outcomes (16). Recent studies have highlighted the involvement of neuronal cell vesicles in various cancers, including gastric cancer, glioma, and head and neck squamous cell carcinoma (15, 17, 18). Moreover, vesicle trafficking has been shown to impact cancer cell drug resistance by modulating the immune microenvironment (19–21). Neuronal vesicle trafficking associated 2(NSG2), also known as HMP19, is localized in the Golgi apparatus of neural and neuroendocrine cells and plays a role in nerve signal transmission (22). Overexpression of NSG2 in primary bone marrow cells has been linked to the proliferation of immature cells and shows promise in inhibiting the growth and spread of pancreatic cancer (23).

This study investigates NSG2’s role in BC by analyzing its immunological and prognostic significance through multiplex immunofluorescence, exploring its correlation with clinical features, outcomes, TIICs, and immune checkpoints, and suggesting its potential as promising indicator for prognosis and immunotherapy target in this disease.

## 2 Materials and methods

### 2.1 Genomic data assessment

Sangerbox database (http://sangerbox.com/Tool) was used for assessing NSG2 expression levels in human BC tissues with those in unpaired normal tissues (24), and Kaplan-Meier Plotter (http://kmplot.com/analysis/) was used to determine NSG2 expression prognostic significance, which determines optimal cutoff values for group categorization.

### 2.2 Clinical tissue samples

BC tissue samples (n = 228) and non-cancerous tissue samples (n = 51) were collected from Nantong Tumor Hospital Affiliated to Nantong University, between 2010 and 2016. Clinical data were retrieved from the hospital’s electronic records, and tissue microarrays (TMAs) were constructed by the Department of Clinical Biobank. No cases underwent preoperative therapy. OS was considered the time from surgery to death or last follow-up. Ethics approval (number 2024-031) was provided by the local hospital’s Human Research Ethics Committee.

### 2.3 Tissue microarray and multiplex immunohistochemical staining

TMA slides were de-paraffinized, rehydrated, and subjected to antigen retrieval before being stained with primary and secondary antibodies using the PerkinElmer Opal 7-Color Technology Kit, DAPI, and scanned at 20× magnification with Vectra 3.0 and analyzed using inForm software.

Cytokeratin (CK)-positive tumor areas were identified to distinguish cancer cells from stromal cells. TIIC levels were quantified using a machine-learning algorithm, and NSG2 protein expression was compared with immune cell types in the BC TMA. The antibodies used for mIHC analysis are shown in **Supplementary Table S1**.

### 2.4 Statistical analysis

GraphPad Prism (version 5.0, USA) and SPSS (version 24.0, USA) were used for data analysis. Pearson χ² tests assessed correlations between NSG2 expression and clinicopathologic features, Cox regression models and Kaplan-Meier curves analyzed survival, and the Spearman test evaluated relationships between TIIC abundance, immune checkpoint expression, and NSG2 levels, using *P* < 0.05 for denoting significant difference.

## 3 Results

### 3.1 NSG2 in BC and prognostic implications

**Figure 1:**
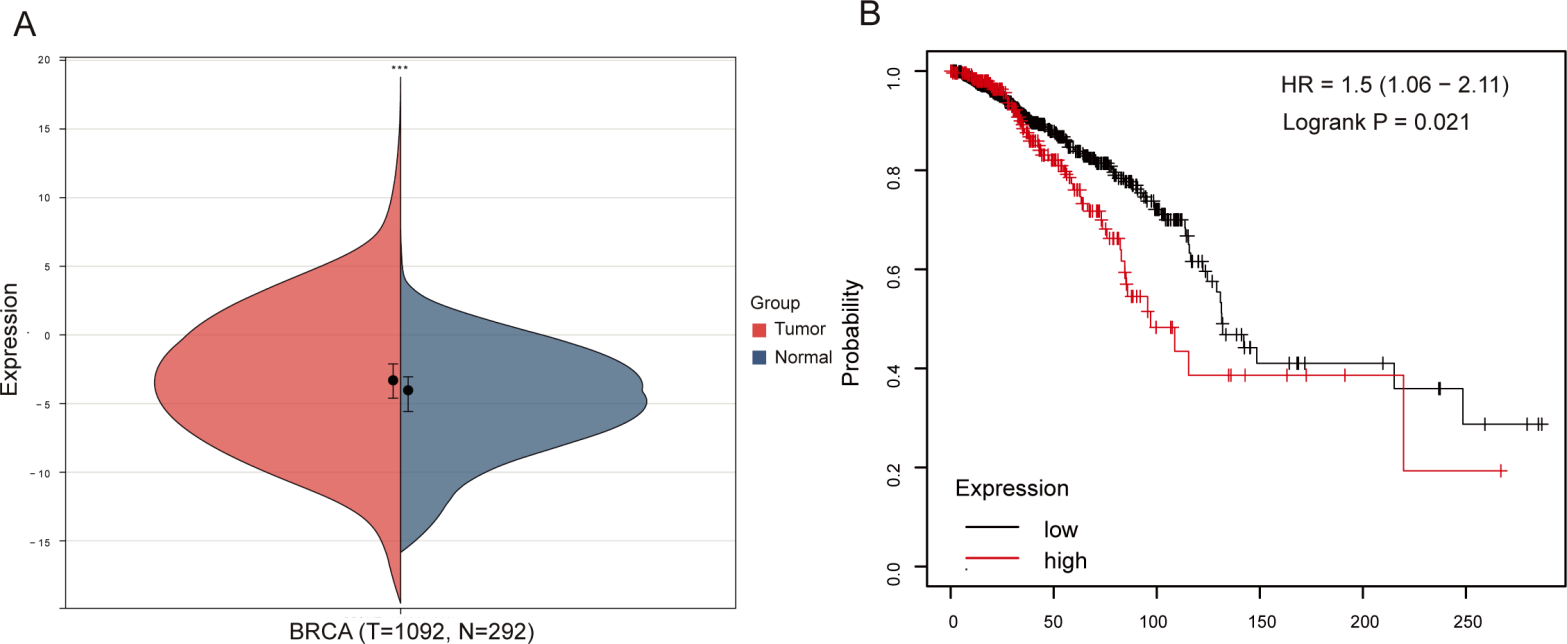
Association between NSG2 expression and OS in breast cancer. **(A)** NSG2 mRNA expression in breast carcinoma versus normal tissues. **(B)** Kaplan-Meier survival curves for high- and low-expression groups.

NSG2 mRNA expression was remarkedly increased in BC tissues (mean ± SEM: -3.35 ± 3.03) than in non-cancerous tissues (mean ± SEM: -4.81 ± 2.71; *P* < 0.05) (**Figure 1A**). Kaplan-Meier survival analysis revealed high tumoral NSG2 expression associated with poorer prognosis (**Figure 1B**).

### 3.2 NSG2 protein expression in BC and non-cancer tissues

**Figure 2:**
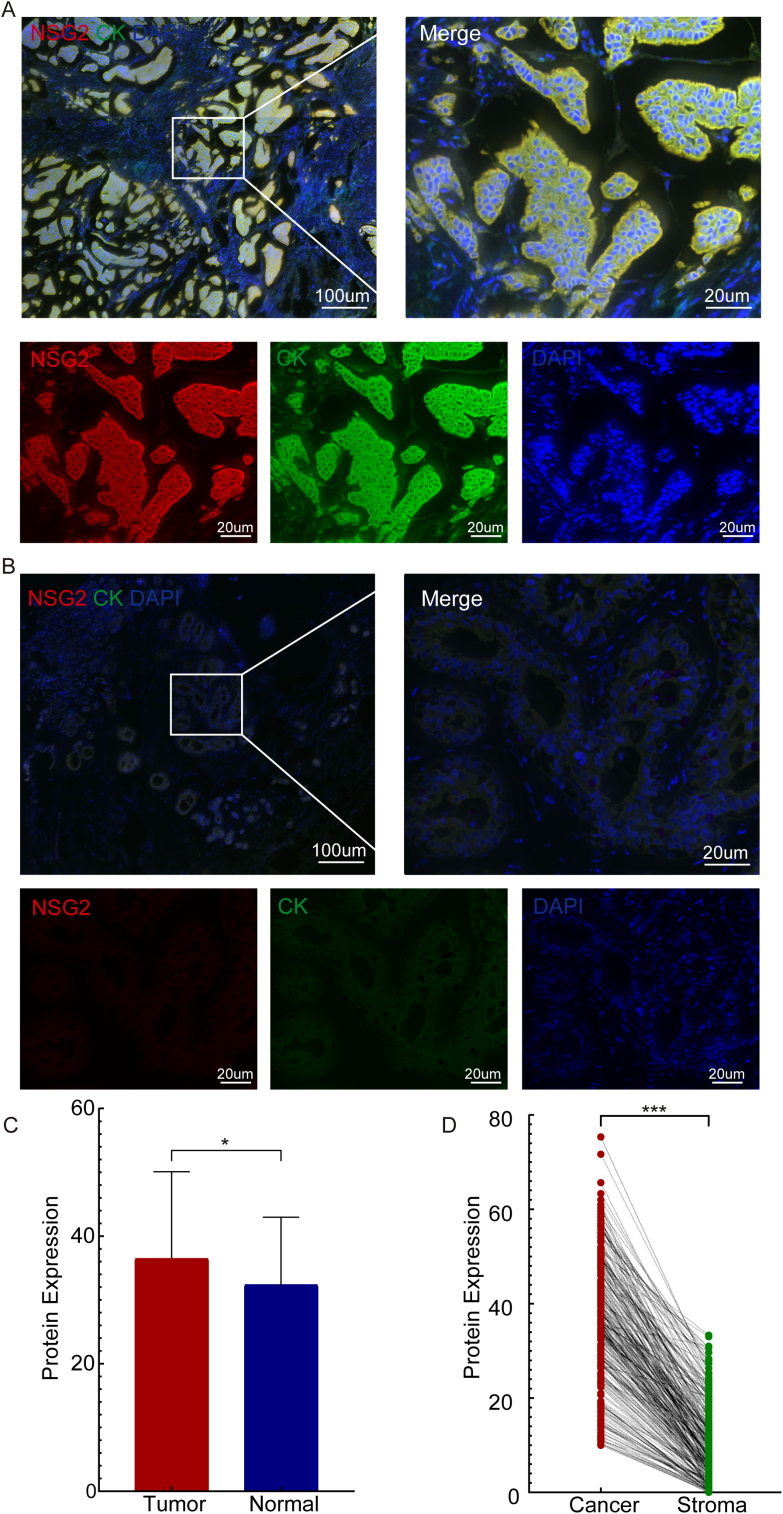
NSG2 protein expression. **(A)** In breast carcinoma tissues. **(B)** In non-cancerous samples. **(C)** Comparison between breast carcinoma and normal tissues. **(D)** Comparison between intratumoral and stromal cells.

Due to post-transcriptional regulatory mechanisms, mRNA expression does not always coincide with the expression of the corresponding protein (25). To evaluate NSG2 protein expression, multiplex immunohistochemistry was used, revealing higher levels in cancer cells (36.47 ± 13.61) compared to ductal epithelial cells (32.36 ± 10.59; Z = -2.003, *P* = 0.045) (**Figures 2A, B**) and greater expression in breast cancer tissues than benign tissues (**Figure 2C**). NSG2 protein was also more prevalent in intratumoral cells than in stromal cells (36.47 ± 13.61 vs. 10.25 ± 8.18; *P* < 0.001) (**Figure 2D**).

### 3.3 Correlation of NSG2 protein expression with clinical features in breast cancer

We determined the optimal cutoff value of NSG2 protein expression in cancer cells using the R package MaxStat. Based on this, we categorized patients into two groups: high NSG2 expression (≥ 33.33, n = 86) and low NSG2 expression (< 33.33, n = 142). For NSG2 protein expression in stromal cells, a cutoff of 16 was used, dividing patients into lower expression (≤ 5.33, n = 83) and higher expression (> 5.33, n = 145) groups. Pearson χ² tests indicated that high NSG2 expression in cancer cells was associated with larger tumor size (*P* = 0.034), distant metastasis (*P* = 0.036), and advanced clinical stage (*P* = 0.032). Conversely, NSG2 expression in TIICs was specifically associated with distant metastasis (*P* = 0.042) (**Table 1**).

**Table 1:** Relationship between NSG2 expression and clinicopathological features.

		NSG2 in cancer cells			NSG2 in stroma cells		
Characteristics	Total	High Expression (%)	Pearson χ2	*P*	High Expression (%)	Pearson χ2	*P*
Total	228	142 (62.3)			145 (63.60)		
Age			0.078	0.780		0.013	0.909
≤ 50	106	65 (61.3)			67 (63.2)		
> 50	122	77 (63.1)			78 (63.9)		
Molecular subtypes			3.319	0.506		0.663	0.956
Luminal A	73	49 (67.1)			49 (67,1)		
Luminal B	58	34 (58.6)			36 (62.1)		
HER2+	17	13 (76.5)			11 (64.7)		
Triple-negative	29	17 (58.6)			18 (62.1)		
Unknown	51	29 (56.9)			31 (60.8)		
T Stage			8.695	0.034^*^		4.315	0.229
Tis+T1	92	51 (55.4)			55 (59.8)		
T2	113	72 (63.7)			73 (64.6)		
T3+T4	11	11 (100)			10 (90.0)		
Unknown	12	8 (66.7)			7 (58.3)		
N Stage			6.748	0.150		6.741	0.150
N0	73	38 (52.1)			42 (57.5)		
N1	62	44 (38.6)			46 (74.2)		
N2	34	23 (67.6)			19 (55.9)		
N3	32	22 (19.9)			23 (71.9)		
Unknown	27	15 (55.6)			15 (55.6)		
M Stage			4.374	0.036^*^		4.134	0.042^*^
M0	221	135 (61.1)			138 (62.4)		
M1	7	7 (100)			7 (100)		
Clinical Stage			8.804	0.032^*^		1.673	0.643
0+I	38	16 (42.1)			21 (55.3)		
II	85	55 (64.7)			57 (67.1)		
III+IV	71	50 (70.4)			46 (64.8)		
Unknown	34	21 (61.8)			21 (61.8)		

**Figure 3:**
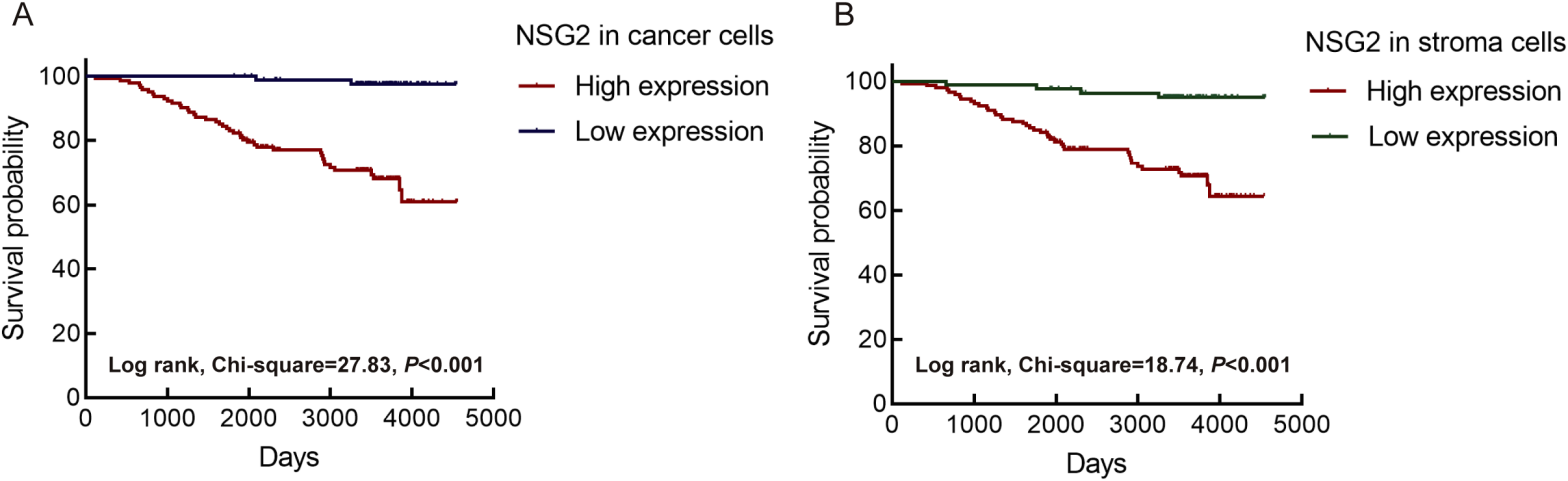
Association between NSG2 protein expression and overall survival. **(A)** Intratumoral cells. **(B)** Stroma cells.

Univariate Cox regression of 228 patients showed that high NSG2 protein expression in both cancer and stromal cells, along with T stage, N stage, M stage, and clinical stage, significantly correlated with OS. Multivariate analysis confirmed NSG2 expression in cancer and stromal cells as independent prognostic factors for poor outcomes (**Table 2**). Kaplan-Meier curves indicated that patients with elevated NSG2 in both intratumoral cells and TIICs had notably worse outcomes (**Figures 3A, B**).

**Table 2:** Univariate and multivariable analyses for OS predictors in BC patients.

Characteristics	Univariate analysis		Multivariate analysis	
	HR (95%CI)	*P* value	HR (95%CI)	*P* value
NSG2 in cancer cells				
High vs. Low	16.611 (4.020-68.647)	< 0.001^*^	8.956 (2.058-38.985)	0.003^*^
NSG2 in stroma cells				
High vs. Low	7.021 (2.512-19.618)	< 0.001^*^	3.092 (1.067-8.957)	0.038^*^
Age (years)				
≤ 50 vs. > 50	1.288 (0.712-2.329)	0.402		
T stage				
Tis+T1 vs. T2 vs. T3+T4	3.092 (1.873-5.104)	< 0.001^*^		
N stage				
N0 vs. N1 vs. N2 vs. N3	1.345 (1.030-1.755)	0.029^*^		
M stage				
M0 vs. M1	4.373 (1.557-12.280)	0.005^*^		
Clinical stage				
I vs. II vs. III+IV	1.753 (1.113-2.276)	0.016^*^	1.539 (0.948-2.499)	0.081
Molecular subtype				
Luminal A vs. Luminal B vs. Her-2 + vs. Tripple negative	1.104 (0.819-1.489)	0.516		

*P<0.05.

### 3.4 NSG2 expression is associated with the abundance of TIICs and immune checkpoints in breast cancer

**Figure 4:**
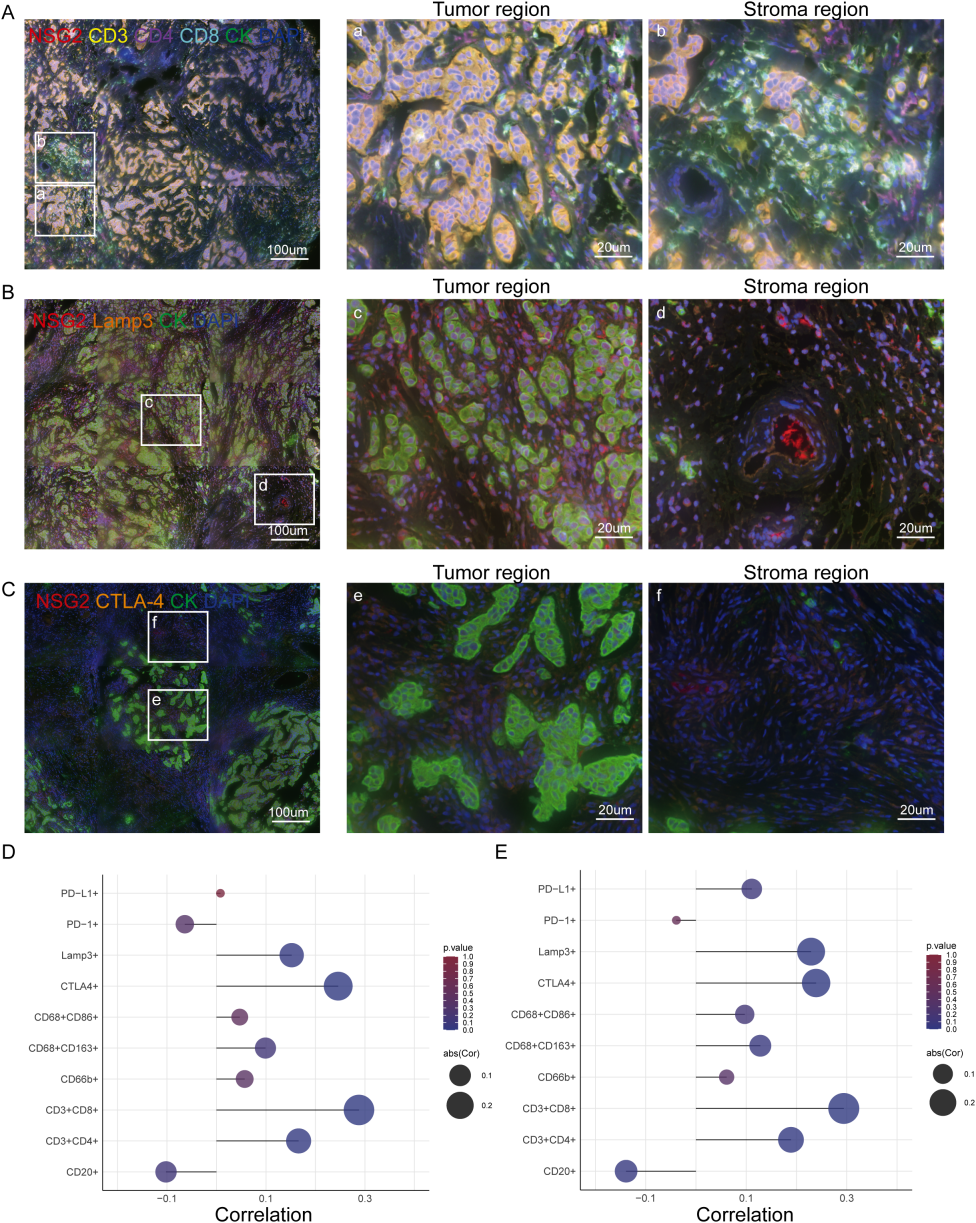
NSG2 expression and its correlation with TIICs and immune checkpoints in BC. **(A)** Six-color multispectral composite images of NSG2, CD3, CD4, CD8, CK and DAPI. **(B)** Four-color multispectral composite images of NSG2, LAMP3, CK and DAPI. **(C)** Four-color multispectral composite images of NSG2, CTLA-4, CK and DAPI. **(D)** Correlation of NSG2 protein expression in cancer cells with immune markers. **(E)** Correlation of NSG2 protein expression in stroma cells with immune markers.

NSG2 expression positively correlated with CD4+ T cells, CD8+ T cells, and Lamp3+ dendritic cells in both cancer and stromal compartment, but negatively correlated with CD20+ B cells in stromal cells (**Figure 4**; **Table 3**). Additionally, NSG2 levels positively correlated with CTLA-4, but showed no significant associations with PD-1 or PD-L1 (**Figure 4**; **Table 3**).

**Table 3:** Association between NSG2 proteins and TIICs and immune checkpoints.

Immune markers	NSG2 in cancer cells		NSG2 in stroma cells	
	r	*P*	r	*P*
CD4+ T	0.166	0.018^*^	0.189	0.007^*^
CD8+ T	0.288	< 0.001^*^	0.294	< 0.001*
CD86+ Macrophages	0.047	0.491	0.097	0.154
CD163+ Macrophages	0.099	0.144	0.128	0.060
CD20+	-0.101	0.138	-0.139	0.041^*^
Lamp3+	0.152	0.024^*^	0.229	< 0.001*
CD66b+	0.057	0.408	0.061	0.376
PD-1	-0.064	0.356	-0.039	0.570
PD-L1	0.008	0.912	0.111	0.109
CTLA-4	0.246	< 0.001*	0.239	< 0.001*

*P<0.05.

## 4 Discussion

Breast cancer continues to be the most widespread malignancy affecting women worldwide, representing a major challenge to patient survival (26). The identification of innovative therapeutic targets is essential for improving clinical outcomes (27). TCGA and GTEx data analysis reveal elevated NSG2 mRNA in BC tissues, with Kaplan-Meier analysis linking high NSG2 levels to poor prognosis, highlighting its potential as a prognostic biomarker.

Our mIHC analysis indicated that NSG2 expression correlates with larger tumor size, distant metastasis, and advanced stage, suggesting its potential as a prognostic marker in breast cancer.

TIICs are important for carcinogenesis (28–30). Our study found that NSG2 expression positively correlates with CD4+ T, CD8+ T and Lamp3+ dendritic cells levels in both cancerous and stromal tissues. Increased NSG2 expression and immune cell infiltration had poorer outcomes. Lamp3+ dendritic cells, known for their role in modulating tryptophan metabolism and exerting immunomodulatory effects, contribute to tumor escape and progression (31, 32). High NSG2 expression areas also showed elevated Lamp3+ dendritic cell levels, correlating with worse outcomes. Additionally, CD4+ and CD8+ T cells are often found at tumor margins (33). Our findings indicate that NSG2 overexpression is associated with increased infiltration of CD4+ and CD8+ T cells near the tumor. CD4+ T cells enhance tumor antigen presentation through interactions with antigen-presenting cells like dendritic cells (34, 35), thereby boosting the cytotoxic activity of CD8+ T cells. Although CD8+ T cells are well-documented for their direct tumor-killing abilities (33, 36, 37), our results suggest that NSG2 may amplify tumor immunogenicity, leading to increased CD4+ and CD8+ T cells populations. This hypothesis warrants further investigation.

In addition, the role of tumor-infiltrating B lymphocytes within the tumor immune microenvironment is increasingly recognized (38). B cells function as antigen-presenting cells, activating CD4+ T and CD8+ T cells to directly target tumor cells (39). The enrichment of CD20+ B cells has been correlated with favorable overall survival (OS) in various solid tumors, including esophageal squamous cell carcinoma and ovarian serous cystadenocarcinoma (40). Specifically, in triple-negative breast cancer, higher levels of CD20+ B cells are associated with improved prognosis (41). Our study demonstrated that elevated NSG2 expression in the stroma correlates with reduced CD20+ B cells presence and poorer prognosis, consistent with previous findings. Notably, this association was observed only in stromal cells and not in intratumoral cells. Given that NSG2 expression is significantly higher in cancer cells than in stromal cells, further verification of the relationship between NSG2 and CD20+ B cells is warranted.

Immune checkpoint inhibitors have shown promise in treating breast cancer (42). CTLA-4, expressed on CD4+ and CD8+ T cells, serves as a critical immune checkpoint regulating T cell activation and proliferation (43). Elevated CTLA-4 levels are associated with diminished immune activity. Targeting CTLA-4 with inhibitors such as ipilimumab has garnered attention as a therapeutic strategy in breast cancer (44). By blocking CTLA-4, these inhibitors enhance T cell responses to tumor cells, thereby potentially enhancing antitumor immunity (45). Our findings indicate a positive correlation between NSG2 and CTLA-4 expression, with higher NSG2 levels linked to poorer prognosis, aligning with existing literature. Despite advancements in CTLA-4 inhibitors, low cure rates and emerging challenges underscore the need for novel treatment strategies (46). Despite advancements in CTLA-4 inhibitors, low cure rates and emerging challenges underscore the need for novel treatment strategies. Our study suggests that NSG2 levels could serve as a predictive biomarker for the efficacy of anti-CTLA-4 immunotherapy, highlighting its potential as a target for future immunotherapeutic approaches.

Nevertheless, this study has several limitations. Prospective validation of these findings is essential. The single-center design may limit the generalizability of our conclusions. Additionally, further validation through cytological and preclinical studies is needed to elucidate the mechanisms by which NSG2 influences BC progression.

In conclusion, this study is the first to identify elevated NSG2 expression in breast cancer, which is linked to poor survival and associated with immune cell abundance and checkpoint expression. NSG2 may thus be a key prognostic and immunological biomarker for breast cancer.

## Data Availability

The raw data supporting the conclusions of this article will be made available by the authors, without undue reservation.
